# Warm Bath in Delirium Tremens

**Published:** 1830

**Authors:** T. H. Wright

**Affiliations:** Physician to the Balt. Alms-house; Baltimore


3. *Observations on the treatment of Delerium Tremens, and on the use of the warm bath in that disease.* By T. H. Wright, M. D. Physician to the Balt. Alms-house.

The treatment, antecedent to the Spring of 1827, in cases complicated with gastric and bibrary derangement, or with much febrile excitement, consisted in the administration of emetics, and afterward, of mild cathartics followed by anodynes graduated in force and repetition, by the amount or obstinacy of the nervous excitement. In some cases of high vascular excitement, the solution of tartar emetic was employed as a nauseant, concurrently with the anodynes. Where sensorial and nervous irritability were greatly exalted, all evacuants were omitted, and the opiate immediately resorted to, but the doses were here made smaller, than where the vital power was less deficient, and in some severe cases of this kind, they were assisted by venous or spiritous potations.

The form of the opiate was sometimes an object of considerable importance. Dovers powder would appear calculated to meet several indications. But independent of its being objectionable from its nauseating properties, it frequently appeared to conduce less to sleep, than the same amount of pure opium. The principal reliance was pure dry opium, the older the better, if well kept. Experience however has taught the advantage of varying the preparation. In one case where sixteen grains of opium has been given in twelve hours without the slightest relief, fifteen drops of black drop procured sleep, in thirty minutes, which lasted fourteen hours.

The sedative powers of the web of the black spider have been fairly tested, and for the most part with partial or doubtful success. In one case, attended with great restlessness, and mental excitement, and with constant loco-motion, in which opium, opium and camphor, the black drop had been given, until the amount approached the utmost limit of safe administration, the web was given on the morning of the third day, 5 grs. every hour. Its effects were prompt and unequivocal. The patient calmed, sensibly to himself, with every dose, and anxiously watched the hour of its repetition. By evening he was so far composed as to consent to lie down, and with the assistance of an opiate, slept continuously for eight hours. The cure was completed by diminished doses of the same medicine on the next day, quiet, suitable nourishment, and an anodyne repeated in the evening.

Hitherto these means had been uniformly successful, but three cases which terminated unsuccessfully in the spring of 1829, tended to weaken the reporters confidence in this mode of treatment, and induced him to look out for some auxiliary, that might enable them to dispense with such large doses of opium. Emetics and mild cathartics were still administered if necessary, and in some cases cupping to the epigastrium, temples, &c. were resorted to as preliminary measures. The warm bath was next used, its temperature regulated by the state of the patient, as a general rule about 90, and its duration to be determined by its effects; always contemplating manifest calming influence.

After the bath, opium (or some of its preparations in the proportion of one grain to the dose) was given every two hours, repeated if necessary to the extent of three or at most four doses. If this did not succeed, the warm bath was repeated and the patient kept in it until sensibly relaxed. The opiate was then renewed in doses of half a grain, repeated every hour until sleep was produced.

Dr. W. speaks with great confidence of the use of the warm bath, and remarks, that after its repetition the reduced doses frequently manifested a greater composing effect than the full doses did before. The students of the institution at length adopted the practice of giving the opiate in small doses, at the commencement, and continuing them until the end was accomplished. The dose now prescribed is ten drops of laudanum at intervals of 40 or 60 minutes, rarely increased, whatever may be the result, to 20 drops. Cases have occurred in which the excitement has continued for two or three days, and at length happily yielded to this course. The bath is generally very agreeable to the feelings of the patient, and they are anxious for its repetition.

Dr. W. is very much opposed to unnecessary restraint, being convinced that it frequently tends to protract the delirious excitement. In confirmation of this view, he relates one protracted case, in which the patient consented to lie if the restraint was removed, and was reported to have fallen asleep in a few minutes. Experience has therefore suggested to him the propriety of the physicians contenting himself, with removing out of the patients way every thing with which he could injure himself, and in severe cases confining him by a leg chain, to a small space near the bed. The opiate is generally exhibited in wine whey, which is also allowed as a drink, unless contra indicated by the grade of excitement. Spirituous drink of all kind is excluded from the plan of treatment.

The following remarks, upon a subject in which every friend of humanity feels a lively concern, will not be read without interest.

When first entering on hospital practice, where the temulent form of disorder was likely to be frequently encountered, it became a question with me whether I might safely withhold spirituous drink from the subjects of that affection betraying a predisposition to the disease in its mature stage, namely, prepared to lapse into agitative delirium. The experiment seemed both morally and medically worth making, if it could be done with safety. It was tried and succeeded. With very rare exceptions, liquor, spirits, wine, &c. was kept wholly out of use, and the temulent subject treated by rest, (sometimes confinement to bed for a day or two,) with sufficient allowance of light nutritious food, and generally a small anodyne at night. I have seen no reason to consider this course unsafe, or calculated to give temulent irritation occasion or opportunity of serious aggravation. Very many who come into the wards with bodies agitated and minds somewhat distempered by temulence, on being put to bed, made warm and comfortable, supplied with suitable and sufficient nourishment, and, if required, a medicinal cordial or anodyne, have tranquillized readily, and escaped the further dominion or the full establishment of the disorder. On the whole, I am sincerely convinced that the administration of spirituous drink is not generally necessary or beneficial in temulence, either as a preventive or remedial mean.

On the subject of the medicinal employment of spirituous liquors in general hospital practice, our experience sustains a conclusion of some interest, especially as it relates to the moral and economical aspect of hospital regimen. Our annual summary of cases treated, for twelve months, ending 1st of May, 1828, gives 1873 patients. A similar estimate for the year ending 1st of May, 1829, furnishes 2167 cases. By a report of the agent of supplies for the Alms-house, the whole quantity of liquors ordered for the institution during those two years, was as follows :—

"For the year ending 1st of May, 1828, 50 gallons of wine, 3 barrels of whiskey, 8 gallons of Jamaica spirits, 1 gallon of gin, 1 dozen bottles of porter. For the year ending 1st of May, 1829, 48 gallons of wine, 2 barrels of whiskey, 7 gallons of gin, 6 gallons of spirits, 2 gallons of French brandy.

"T. H. HAND, *Agent.*"

The "whiskey" mentioned in the report of the agent, was used for various purposes, in the house, chiefly stimulant fomentations, liniments, &c. and for making common tinctures. None of it was administered to patients, except as made into tinctures. There remains then, of wine, spirits, brandy, &c. a total of one hundred and twenty-eight gallons used in two years, with an aggregate of patients in the time amounting to four thousand and forty. In a professional view, the first question which ought to associate itself with such an exhibit, is whether the subjects and diseases of hospital practice will safely admit so restricted employment of stimulant means. If my judgment and experience have not both greatly deceived me, I can conscientiously answer in the affirmative. My real conviction is, that what error we committed in the use of those agents was on the side of indulgence rather than restriction—that we gave too much instead of too little. The total mortality in the institution, for the two years indicated, was 322, number of cases 4040, ratio of deaths about twelve and a half per cent. A result it is conceived not less favourable than has obtained in any similar establishment abroad or at home. The ratio of mortality here given, will be greatly reduced in a medical contemplation, by the fact that the aggregate of deaths reported comprehends one hundred and two cases of pneumo-tuberculitis, or chronic phthisis pulmonalis.

*Baltimore, December*, 1829.

As the reported is disposed to congratulate himself, very justly we think, upon the success of his practice we will remark that success is greater than he has himself represented it, 322 out of 4040 being eight instead twelve and a half per cent.

